# Gallbladder Volvulus: A Rare Entity with “Beak Sign” and “Whirl Sign”

**DOI:** 10.5334/jbsr.4030

**Published:** 2025-08-21

**Authors:** Margaux Vlasselaer, Martina Pezzullo, Gabriel Liberale

**Affiliations:** 1 Department of Radiology, Erasme Hospital, ULB, Route de Lennik 808, 1070 Bruxelles, Belgium; 2 Department of Surgery, Erasme-Bordet Hospital, HUB, Route de Lennik 808, 1070 Bruxelles, Belgium

**Keywords:** gallbladder, volvulus, cholecystitis, elderly, whirl sign, bird’s beak sign, ischemia

## Abstract

*Teaching point:* Gallbladder volvulus is a rare but serious diagnosis that needs urgent surgical treatment to avoid morbidity and mortality related to gallbladder ischemia and should be suspected in elderly female patients with acute right upper quadrant pain with imaging features including enlarged, abnormally positioned gallbladder and specific key findings on CT including “whirl sign” representing twisting of the cystic pedicle and “beak sign” at the gallbladder neck.

## Case History

A 95-year-old female patient was referred to the emergency department for acute right upper quadrant pain lasting for one week associated with a palpable mass without fever, jaundice, nausea, or vomiting. Laboratory results showed a mild inflammatory syndrome (CRP 64 mg/L, WBC 13,700/mm³) and normal liver function tests with slightly conjugated hyperbilirubinemia (0.4 mg/dL).

**Figure 1:**
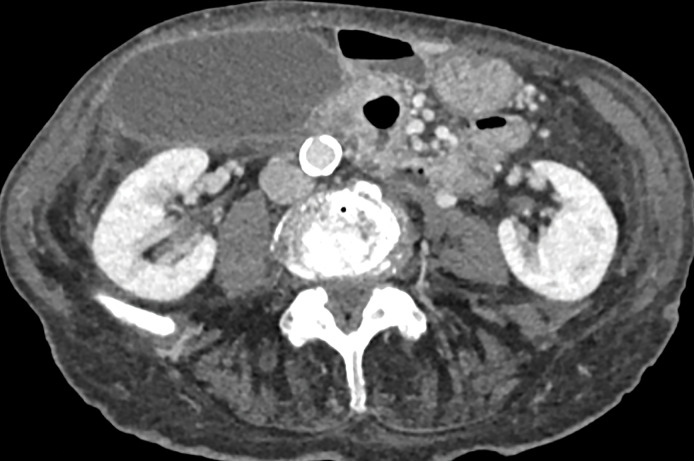
Axial image of a CT scan during the portal phase showing distended gallbladder in horizontal position, outside the liver bed, with no dense gallstones and mild pericholecystic fluid.

**Figure 2:**
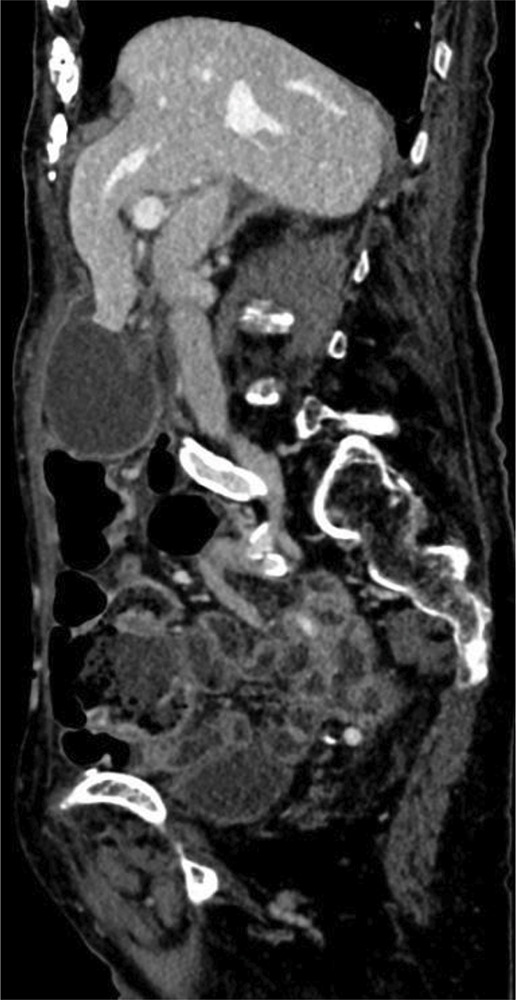
Sagittal image of a CT scan during the portal phase showing distended gallbladder inferior to the liver with “whirl sign” involving the cystic pedicle and “beak sign” with the distended gallbladder tapering at the gallbladder neck.

**Figure 3:**
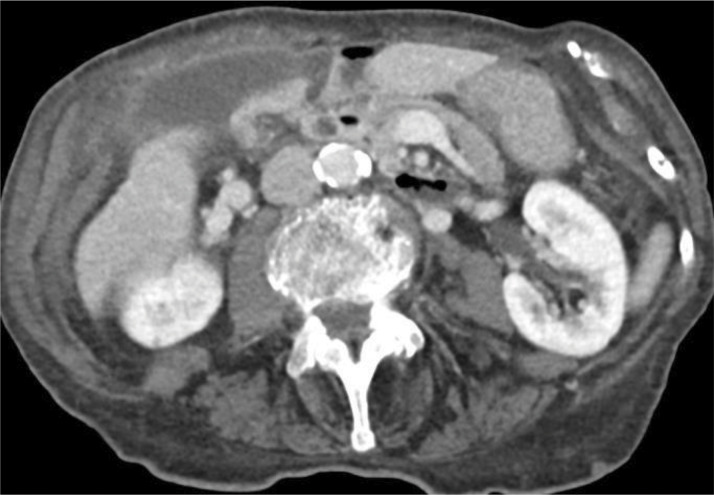
Axial image of a CT scan during the portal phase most obviously showing the “whirl sign” representing twisting of the cystic pedicle and “beak sign” at the gallbladder neck.

**Figure 4:**
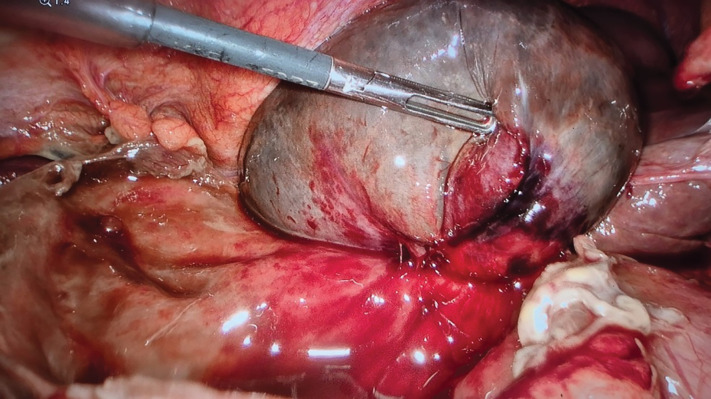
Intra-operative photograph during laparoscopy revealed a complete double spiral torsion of the gallbladder around the cystic duct and artery, confirming the diagnosis of gallbladder volvulus.

Abdominal computed tomography (CT) was performed for suspicion of a neoplasia given the context (no ultrasound performed). The contrast-enhanced CT showed a distended gallbladder in an unusual horizontal position, outside the liver bed and inferior to the liver, with no dense gallstones (Figures 1 and 2). A “whirl sign” involving the cystic pedicle (cystic duct and cystic artery) and a “beak sign” with the distended gallbladder tapering at the pedicle were observed (Figures 2 and 3). Mild pericholecystic fluid was present (Figures 1 and 3). The diagnosis of gallbladder volvulus was suggested. Surgical laparoscopic exploration revealed a complete double spiral torsion of the gallbladder around the cystic duct and artery, confirming the diagnosis of gallbladder volvulus (Figure 4). The gallbladder was removed after detorsion. The patient recovered uneventfully.

## Comments

Gallbladder volvulus is an uncommon but critical condition, occurring most often in elderly women mimicking acute cholecystitis and usually associated with progressive weight loss [1]. Early imaging diagnosis is crucial, as the condition progresses to gangrene and perforation if untreated [1]. Ultrasonography is often the first-line imaging modality revealing an enlarged, thick-walled gallbladder located in an aberrant position, often horizontally and inferior to the liver [1]. Doppler ultrasound may show interrupted blood flow in the cystic pedicle [1]. The rotation of the gallbladder around the axis of the cystic duct and cystic artery explains the radiological key findings on CT, including “whirl sign” representing twisting of the cystic pedicle and “beak sign” at the gallbladder neck [1]. Magnetic resonance cholangiopancreatography offers the advantage of revealing the cystic duct and is an excellent tool for detecting gallbladder volvulus (twisted cystic duct) in atypical or equivocal cases [1]. Torsion of the vascular pedicle can lead to ischemia, necrosis, and perforation [1]. Early recognition of gallbladder volvulus on imaging is crucial, especially when the clinical context is atypical for classical cholecystitis. Management is prompt cholecystectomy [1].
